# Increased Respiratory Syncytial Virus-Associated Hospitalizations and Ambulatory Visits in Very Preterm Infants in the First Year of Life following Discontinuation of Access to Palivizumab

**DOI:** 10.1055/a-2512-9453

**Published:** 2025-01-31

**Authors:** Yolanda Evong, Jiaxin Luo, Lingyun Ye, John Fahey, Janis L. Breeze, Rebecca Attenborough, Kenny Wong, Joanne M. Langley

**Affiliations:** 1 Department of Pediatrics, Dalhousie University Faculty of Medicine, Izaak Walton Killam Health Centre, Halifax, Nova Scotia, Canada; 2 Canadian Center for Vaccinology (Dalhousie University, IWK Health and Nova Scotia Health), Halifax, Nova Scotia, Halifax, Nova Scotia, Canada; 3 Canadian Centre for Vaccinology, Dalhousie University, Halifax, Nova Scotia, Canada; 4 Department of Reproductive Care of Nova Scotia, IWK Health Centre, Halifax, Nova Scotia, Canada; 5 Division of Pediatric Cardiology, Department of Pediatrics, Dalhousie University Faculty of Medicine, IWK Health Centre, Halifax, Nova Scotia, Canada; 6 Department of Pediatrics and Community Health and Epidemiology, Dalhousie University, Halifax, Nova Scotia, Canada

**Keywords:** infant, premature, antibodies, monoclonal, respiratory syncytial virus, health policy

## Abstract

**Objective:**

From 2002 to 2023, palivizumab was the only intervention to reduce respiratory syncytial virus (RSV)-associated hospitalizations in high-risk infants in Canada but advances in RSV prevention are drastically changing this landscape. Eligibility criteria for this monoclonal antibody for preterm infants varied over time across each of 10 Canadian provinces and 3 territories. The National Professional Pediatric Association (Canadian Pediatric Society) revised its eligibility recommendations in 2015, removing access for preterm infants 30 to 32 weeks gestation (WG). The province of Nova Scotia followed these recommendations the next season. This study aimed to determine if the removal of access to palivizumab in these previously eligible infants was associated with a change in hospital admissions, deaths, or ambulatory visits associated with RSV.

**Study Design:**

We identified a retrospective cohort of Nova Scotia infants born between 30 and 32 WG, without other risk factors for RSV-H, from April 2012 to September 2019 by linking six population-based provincial databases, and followed each infant through the first year of life. Episodes of RSV-associated hospitalization (RSV-H), ambulatory visits (RSV-A), or death were identified by the International Statistical Classification of Diseases and Related Health Disorders (ICD) RSV-associated diagnostic codes.

**Results:**

Of 4,835 infants born during the study period, 250 were 30 to 32 WG and eligible for the cohort. RSV-H increased approximately 10-fold following restricted access to palivizumab (from no RSV-H (0/123) to 9.4%; 95% CI 5.0, 15.9; risk difference 9.4), but no RSV-associated deaths occurred. RSV-A also increased from 5.7 to 17.3% (risk difference 11.6).

**Conclusion:**

Discontinuation of access to a prophylactic anti-RSV monoclonal antibody in very preterm infants was associated with a higher risk of RSV-H and RSV-A. Evaluation of health care policy change on patient well-being is essential to assess the impact and guide future decision-making at the population level.

**Key Points:**

- Discontinuation of access to a prophylactic anti-RSV monoclonal antibody in very preterm infants 30 to 32 WG was associated with a higher risk of RSV-H and RSV-A. Evaluation of changes to health care policy on patient well-being is essential to assess impact and guide future decision-making at the population level.
- Removing access to palivizumab led to higher RSV admissions in 30 to 32 WG infants.
- The effect of health care policy changes on child well-being should be assessed routinely.
- No deaths associated with RSV were identified prior to or after the policy change.

Respiratory syncytial virus (RSV) is the leading cause of hospitalization among infants in the first year of life, with the highest rates in those under 6 months of age. 1 2 From 2002 until 2023, the only intervention to reduce the risk of serious RSV-associated illness in Canadian infants was the anti-RSV F monoclonal antibody palivizumab (Synagis®, AstraZeneca Canada Inc.) administered monthly up to five times during the RSV season. In 2023, a higher efficacy, longer-acting monoclonal antibody (nirsevimab, Sanofi), and a maternal RSV vaccine (RSV-preF, Pfizer) were authorized in Canada, dramatically changing the opportunities for RSV prevention. in 2024, the Canadian National Advisory Committee on Immunization recommended that jurisdictions work toward nirsevimab programs for all infants, depending on feasibility. 3 For the 2024–2025 season, most provinces will not introduce universal nirsevimab programs.

Recommendations for eligibility for palivizumab by jurisdictions varied from 2002 to 2022, generally restricting access over time. In 2014, the American Academy of Pediatrics (AAP) advised that prophylaxis may be administered to premature infants without lung or heart disease born before 29 ^0/7^ weeks gestation (WG) and younger than 12 months at the start of the RSV season. 4 5 In 2015, the Canadian Pediatric Society (CPS) updated its guidance to exclude otherwise healthy very preterm infants born above 30 WG; in 2016, the province of Nova Scotia revised its eligibility criteria to align with this guidance.

As jurisdictions plan and implement their RSV prevention programs, it will be important to assess their impact. In this study, we aimed to determine if the revised provincial policy was associated with a change in the burden of illness associated with RSV in children no longer eligible for prophylaxis, as measured by a change in hospital admissions, deaths, or ambulatory visits associated with RSV.

## Materials and Methods

### Study Design

This was an observational, retrospective cohort study.

### Setting

Nova Scotia is a Canadian province on the Atlantic seaboard, with a population of 1 million, and universal health care.

### Data Sources

The inception cohort of eligible preterm newborns and their RSV-associated illness in the first year of life was assembled by linking six provincial databases. The population-based Reproductive Care Program (RCP) Nova Scotia Atlee Perinatal Database (NSAPD) identified each infant at birth and gestational age. Eligibility criteria were determined using the Nova Scotia Provincial Blood Coordinating Program Palivizumab Database, the IWK Cardiology Database, and the Vital Statistics Database. Outcomes were determined from the Medical Services Insurance (MSI) Database, and the Canadian Institutes for Health Information Discharge Abstract Database (CIHI-DAD).

The data were available as part of a data sharing agreement between the Nova Scotia government Department of Health and Wellness, and two health authorities, Nova Scotia Health and the IWK Health Centre. We obtained Research Ethics Board approval from the IWK Health Centre. As this was a secondary use of existing data on events over two decades, the risk of identification was very low, and parental/guardian consent was not deemed necessary.

### Participant Identification

To identify the eligible cohort, all infants born in Nova Scotia between 30 and 37 WG from April 1, 2012, to September 30, 2019, were identified in the NSAPD. The unique provincial health card number (HCN) was used to link each child to their data in the five other databases. HCNs were substituted with random identifiers prior to export for analysis.

Infants were excluded if their gestational age information was not available, were born after 32 ^0/7^ weeks gestational age (WG), were not a resident of Nova Scotia, died in the neonatal period (<28 days of age), or were eligible for palivizumab prophylaxis due to chronic lung disease or requiring supplemental oxygen within 6 months of the RSV season or had hemodynamically significant congenital heart disease (CHD), or received one or more doses of palivizumab. Each infant was followed for the outcomes of interest from birth throughout the first year of life. The last participant completed their follow-up in 2020.

### Outcomes

The primary outcome measure was RSV-H in the first year of life. RSV-H events were defined as one or more of the RSV-specific International Statistical Classification of Diseases and Related Health Problems (ICD) diagnostic codes (B97.4, J20.5, J21.0, and J12.1) for that child in the CIHI-DAD or the MSI databases.

The secondary outcomes were ambulatory visits to a physician (RSV-A), deaths associated with RSV, and the percentage of children receiving palivizumab over time.

### Data linkage and Analysis

The eligible population was described. The frequency of RSV-H, RSV-A, RSV-associated death, and palivizumab use was described for each of the 9 study years. Risk differences before and after the policy change, with 95% confidence intervals, were calculated. Means were utilized to report the total percentage of infants who received palivizumab prior to and after the policy change.

## Results

### Population

**Fig. 1:**
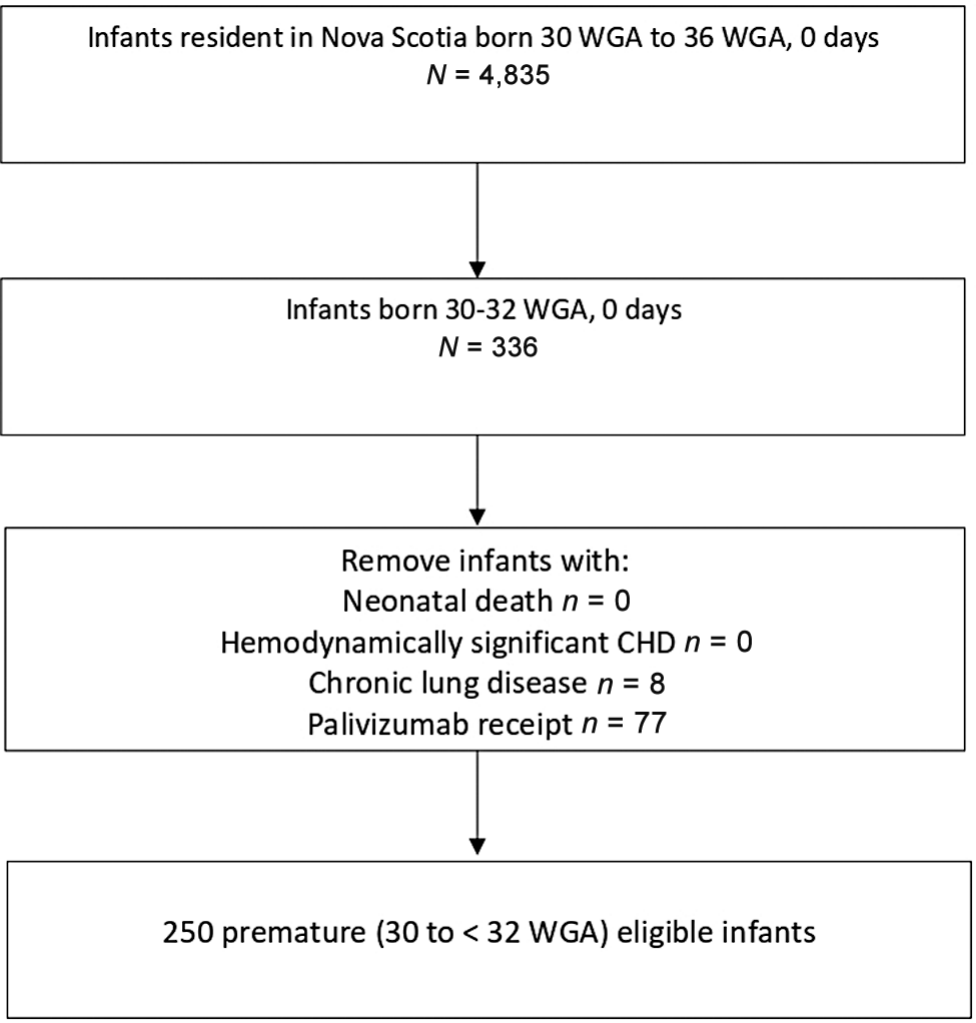
Identification of a cohort of very preterm infants born at 30 to 32 weeks gestation. WGA, weeks of gestational age.

There were 4,835 infants born in Nova Scotia between 30 and 37 WG from April 1, 2012, to September 30, 2019. of these, 337 were born between 30 and 32 ^0/7^ WG ( Fig. 1 ). After excluding infants with hemodynamically significant heart disease and/or lung disease, or receipt of palivizumab, 250 healthy preterm infants constituted the inception cohort.

### Outcomes

#### Respiratory Syncytial Virus-Associated Hospitalizations

RSV-H increased following the policy change, with no admissions prior to the 2016–2017 season and 12 admissions in the first year of life after restricted access to prophylaxis (12/127; 9.4%, 95% CI 5.0, 15.0). The risk difference of RSV-H in the pre- and post-policy periods was 9.4% (12/127 − 0/123 = 0.094, 95% CI 0.04, 0.145), or a nearly 10-fold increase in risk.

#### Respiratory Syncytial Virus-Associated Ambulatory Visits

Ambulatory visits to physicians increased after the introduction of the new policy restricting access to these very preterm infants. In the period prior to the policy change, population with RSV-associated visits occurred in 5.7% (7/123; 95% CI 2.3%, 11.4%) whereas post-policy change, 17.3% of infants (11/127; 95% CI 11.2%, 25.0%) had RSV-associated ambulatory care events, representing a risk difference of 11% (95% CI 0.03, 0.19).

#### Respiratory Syncytial Virus-Associated Mortality

There were no deaths associated with RSV during the study period in the study population.

#### Palivizumab Coverage

Use of palivizumab declined in the cohort after the 2016–2017 season provincial policy change, with 40.9% (72/195) of otherwise healthy preterm infants born between 30 and 32 WG receiving palivizumab between before, and 3.8% (5/132) in receipt after.

## Discussion

In this province-wide, population-based, retrospective cohort study, we found that implementing a recommendation to remove access to RSV prevention with palivizumab in healthy preterm infants of 30 to 32 WG was associated with an increase in RSV-associated hospitalizations and ambulatory visits, but not with mortality. In the RSV seasons following the AAP and CPS guideline changes, several observational studies have shown a decline in palivizumab eligibility is associated with an increase in rates of bronchiolitis, hospitalizations, intensive care use, and ambulatory visits associated with RSV. 6 7 8 Our study adds to the growing body of data that suggest very preterm babies born between 30 and 32 WGA, and with no comorbidities, are at a significantly higher risk of RSV-associated disease significant enough to warrant medical attention.

The results of this study also support recent recommendations from the United States, Canada, and several European countries to offer universal RSV prevention programs with the long-acting monoclonal antibody nirsevimab (Beyfortus®, Sanofi) to infants entering or born during their first RSV season, or maternal RSV vaccine. While making this recommendation in 2024, Canada's National Advisory Immunization Committee recognized that it may not be possible to offer universal programs immediately and recommended prioritizing the highest-risk infants for nirsevimab and using palivizumab according to previous recommendations from 2022. 3 9 We note that the 2022 recommendations exclude infants with 30 WGA and older and therefore urge jurisdictions to carefully consider whether infants 30 ^0/7^ to 32 WGA can be offered prevention in a risk-based nirsevimab or palivizumab program. Based on this study Nova Scotia reversed its 2016 decision and is offering prophylaxis to infants <32 ^0/7^ WG as of the 2023–2024 season.

Physicians and health care providers can use the study results to counsel families with an infant born between 30 and 32 weeks of GA about their risk of severe RSV disease so that parents can make informed decisions about protecting their child during their child's first RSV season. These results may also affect the decision-making of the physicians themselves when caring for this population, and their willingness to refer the patient for an anti-RSV monoclonal antibody. Finally, physicians may choose to advocate for policy change if they practice in a jurisdiction that does not currently offer RSV prevention to these babies.

## Limitations

As this cohort was constructed from secondary datasets, it could be subject to coding limitations and data entry errors. We expect that RSV-associated illness is underreported in our databases as routine virologic testing is not standard of practice for all patients presenting with respiratory symptoms, and therefore the burden of disease is likely underestimated. This is a descriptive study so causality cannot be attributed. The study population was exclusively from Nova Scotian which may limit generalizability to other geographic areas due to differing health care, socioeconomic, and climate circumstances. Finally, studies using secondary data are limited in their ability to control confounders.

## Conclusion

Nova Scotian infants born between 30 and 32 WGA had higher rates of severe RSV disease after becoming ineligible for palivizumab, but no increase in mortality. Evaluation of health care policy is essential to assessing benefits and risks.
